# Du Bois–Reymond Type Lemma and Its Application to Dirichlet Problem with the *p*(*t*)–Laplacian on a Bounded Time Scale

**DOI:** 10.3390/e23101352

**Published:** 2021-10-16

**Authors:** Jean Mawhin, Ewa Skrzypek, Katarzyna Szymańska-Dȩbowska

**Affiliations:** 1 Institut de Recherche en Mathématique et Physique, Université Catholique de Louvain, Chemin du Cyclotron, 2, B-1348 Louvain-la-Neuve, Belgium; jean.mawhin@uclouvain.be; 2 Institute of Mathematics, Lodz University of Technology, Al. Politechniki 10, 93-590 Łódź, Poland; ewa.skrzypek@dokt.p.lodz.pl

**Keywords:** du Bois–Reymond lemma, *p*(*t*)–Laplacian, time scales, variational methods, direct variational method, mountain pass lemma

## Abstract

This paper is devoted to study the existence of solutions and their regularity in the p(t)–Laplacian Dirichlet problem on a bounded time scale. First, we prove a lemma of du Bois–Reymond type in time-scale settings. Then, using direct variational methods and the mountain pass methodology, we present several sufficient conditions for the existence of solutions to the Dirichlet problem.

## 1. Introduction

Variational methods and critical point theory have been very successful in obtaining existence and multiplicity results for nonlinear ordinary or partial differential equations, as well as for nonlinear difference equations submitted to various boundary conditions. See, for example, [1,2,3,4,5] and their references.

The aim of this paper is to use those methodologies for the study of the Dirichlet problem for a dynamic equation on a bounded time scale T involving the p(t)–Laplacian,

(1) $$\begin{array}{c} \left\{\begin{array}{c} -\Delta_{p(t)}u\left(t\right):=-\frac{\Delta }{\Delta t}\left(|\Delta^{w}{u\left(t\right)|}^{p(t)-2}\Delta^{w}u\left(t\right)\right)=f\left(t,u^{\sigma }\left(t\right)\right),\;t\in T\\ u(a)=u(b)=0 \end{array}\right.. \end{array}$$

In this equation, $\Delta^{w}$ denotes a weak derivative operator defined in terms of the Δ–integral on a time scale (see Section 2 for precise definitions), p:T→(1,∞) is a measurable and essentially bounded function with an essential lower bound larger than one, and *f* is a Δ–Carathéodory function.

A partial motivation is the paper of Xian-Ling Fan and Qi-Hu Zhang [6] dealing with a similar problem in the case of a partial differential equation. Such equations are known to describe mathematical models of various phenomena arising in the study of elastic mechanics [7] or image restoration [8]. Early variational approaches on Dirichlet problems with *p*–Laplacian are quoted in [9], extensions to p(x)–Laplacian are given in [6,10], and some generalizations (anisotropic problems) are described in the paper [4]. Since the research was conducted in discrete and continuous settings separately, it seems interesting to demonstrate that a sort of unification is also possible with the use of a time-scale notion considered with some type of measure that has not been vastly exploited but which appears indispensable. For boundary value problems on time scales, one can consult [11]. Since we take the definition of the Δ–measure from [11], it is necessary to provide additional proof regarding the absolutely continuity of functions defined over subsets containing the maximum of the bounded time scale T.

The underlying Lebesgue and Sobolev spaces with variable exponents, where the variational approach takes place, are defined in Section 2, where their required properties are proved. The first paper on the variable exponent Lebesgue and Sobolev spaces $L^{p(x)}\left(\Omega \right)$ and $W^{m,p(x)}\left(\Omega \right)$, $\Omega \subset R^{n}$ is due to Kováčik and Rákosník [12] and was developed in [13]. Some earlier papers on the Lebesgue and Sobolev spaces on time scales are [11,14], and we refer to [15] for further basic information on the variable exponent Lebesgue and Sobolev spaces on time scales.

The variational treatment of problem (1) requires proving a so-called du Bois–Reymond Lemma in this new frame to make the link between the critical points of the action functional and the solutions of the boundary value problem. This is done in Section 3 (Lemma 3). It also requires a careful study of the differentiability and other properties of the action functional. This is the object of Section 4.

We are now ready to apply in Section 5 the direct method of the calculus of variations to prove the existence of a solution to problem (1) when $F\left(t,u\right):=\int_{0}^{t}f\left(s,u\right)\;\Delta s$ is bounded above by an expression of the form $c_{1}\left|u\right|+c_{2}\frac{{\left|u\right|}^{\beta }}{\beta }+c_{3}$, where the $c_{i}$ are positive constants, $c_{2}$ is sufficiently small and $\beta \in (1,{ess inf}_{T}p]$ (Theorem 2). This is the essence of Theorem 2.

When F(t,u) grows faster than ${ess sup}_{T}p$ at infinity, the action functional need not have a minimum, but the simplest of the minimax method, namely the mountain pass lemma, may be used to prove the existence of a nontrivial solution to problem (1) when f(t,0)=0 and f(t,u) is sufficiently ‘flat’ in *u* near u=0. This is done in Theorem 4, where the growth of *F* for large *u* is governed by a suitable Ambrosetti–Rabinowitz condition and f(t,u)→0 when u→0 faster than ${\left|u\right|}^{{ess sup}_{T}p-1}$.

## 2. Variable Exponent Lebesgue and Sobolev Spaces on Time Scales

In this section, we recall some basic facts concerning functions defined on time scales (see [11,14,16,17]) and discuss the variable exponent Lebesgue and Sobolev spaces on time scales (see [15]).

Let T be a bounded time scale. We define

(2) a=inf{s∈T},b=sup{s∈T}.

Since T is bounded, a,b∈T. Define the forward jump operator σ:T→T by

(3) $$\sigma \left(t\right)=\left\{\begin{array}{cc} \inf\{s\in T:s>t\} & \mathrm{for}t\in T\backslash \{b\}\\ b & \mathrm{for}t=b \end{array}\right..$$

If σ(t)>t, then the point t∈T is said to be right-scattered. If σ(t)=t, then t∈T is called a right-dense point. The backward jump operator ϱ:T→T is as follows:

(4) $$\varrho \left(t\right)=\left\{\begin{array}{cc} \sup\{s\in T:s<t\} & \mathrm{for}t\in T\backslash \{a\}\\ a & \mathrm{for}t=a \end{array}\right..$$

If ϱ(t)<t, then we say that the point t∈T is left-scattered. If ϱ(t)=t, the point t∈T is called left-dense.

Let $R_{T}=\left\{t\in T:t<\sigma \left(t\right)\right\}$ and u:T→R. We define the step interpolation $\hat{u}:\left[a,b\right]\to R$ as

(5) $$\begin{array}{c} \hat{u}\left(t\right)=\left\{\begin{array}{cc} u(t) & \mathrm{for}t\in T\\ u(s) & \mathrm{for}t\in \left(s,\sigma \left(s\right)\right),\;s\in R_{T} \end{array}\right.. \end{array}$$

The function $\hat{u}$ extends *u* to [a,b], and it enables us to establish equivalence between Lebesgue Δ–integrable and integrable functions. Function u:T→R is Δ−measurable (Δ−integrable) if $\hat{u}:\left[a,b\right]\to R$ is measurable (integrable) on [a,b] in the Lebesgue sense. We say that $u\in L^{1}\left(T\right)$ if

$$\begin{array}{c} \int_{T}u\left(t\right)\Delta t=\int_{\left[a,b\right]}\hat{u}\left(t\right)dt<\infty . \end{array}$$

$L^{1}\left(T\right)$ is a Banach space with the norm

$$\begin{array}{c} {∥u∥}_{L^{1}\left(T\right)}=\int_{T}u\left(t\right)\Delta t. \end{array}$$

A subset A⊂T is Δ−measurable if its characteristic function $\chi_{A}:T\to R$ is Δ–measurable. We define the notion of Δ−measure $\mu_{\Delta }\left(A\right)$ of A⊂T by

$$\begin{array}{c} \mu_{\Delta }\left(A\right)=\int_{T}\chi_{A}\left(t\right)\Delta t=\int_{\left[a,b\right]}{\hat{\chi }}_{A}\left(t\right)dt, \end{array}$$

where ${\hat{\chi }}_{A}:\left[a,b\right]\to R$ is the extension (see (5)) of the characteristic function $\chi_{A}:T\to R$. The subset A⊂T is called Δ−null set if $\mu_{\Delta }\left(A\right)=0.$

For each $t_{0}\in T\backslash \left\{b\right\}$, the single-point set $\left\{t_{0}\right\}$ is Δ−measurable and $\mu_{\Delta }\left(\left\{t_{0}\right\}\right)=\sigma \left(t_{0}\right)-t_{0}$. For every right-scattered point $t_{0}\in T$, it holds that $\sigma \left(t_{0}\right)>t_{0}$. This implies that $\mu_{\Delta }\left(\left\{t_{0}\right\}\right)>0$ for every $t_{0}\in R_{T}$. In particular, if T is a discrete time scale, then $\mu_{\Delta }\left(\left\{t\right\}\right)>0$ for all t∈T\{b}. Moreover, we know that $\mu_{\Delta }\left(\left\{b\right\}\right)=\int_{\left\{b\right\}}1\Delta t=\int_{\left[a,b\right]}1{\hat{\chi }}_{\left\{b\right\}}\left(t\right)dt=\mu_{L}\left(\left\{b\right\}\right)=0$, where *b* is given in (2) (see [11]) and $\mu_{L}$ denotes the classical Lebesgue measure. Hence, all subsets of the time scale T containing *b* are of a finite Δ−measure, and this is the main difference from the approach given in [14].

Let u:T→R. The continuity of *u* is defined in the usual manner. A function *u* is rd-continuous if it is continuous at every right-dense point and if the left-sided limit exists in every left-dense point. Denote by $C_{\mathrm{rd}}\left(T\right)$ (respectively C(T)) the set of rd–continuous (respectively continuous) functions u:T→R. With the norm

$$\begin{array}{c} {∥u∥}_{T}=\underset{t\in T}{\sup}\left|u\left(t\right)\right|, \end{array}$$

these spaces are Banach spaces.

We denote $u^{\sigma }\left(t\right)=u\left(\sigma \left(t\right)\right)$ for t∈T, where σ is defined in (3). If u∈C(T), then $u^{\sigma }\in C_{\mathrm{rd}}\left(T\right)$. Moreover, one has

(6) $$\begin{array}{c} {∥u^{\sigma }∥}_{T}\leq {∥u∥}_{T}. \end{array}$$

Let us denote $T^{\kappa }=T\backslash \left(\varrho \left(\sup T\right),\sup T\right]$, where ϱ:T→T is defined in (4). In this way, we remove from the time scale T its left-scattered maximum, when necessary. Alternatively, it can be written as

$$\begin{array}{c} T^{\kappa }=\left\{\begin{array}{cc} T & \mathrm{if}b\mathrm{is}\mathrm{not}\mathrm{an}\mathrm{isolated}\mathrm{point},\\ T\backslash \{b\} & \mathrm{if}b\mathrm{is}\mathrm{an}\mathrm{isolated}\mathrm{point}. \end{array}\right. \end{array}$$

We recall that u:T→R is Δ−differentiable at $t\in T^{\kappa }$ if there exists a finite number $f^{\Delta }\left(t\right)$ with the property that given any ε>0, there is a neighborhood U⊂T of *t* such that

$$|u^{\sigma }\left(t\right)-u\left(s\right)-u^{\Delta }\left(t\right)\left(\sigma \left(t\right)-s\right)|\leq \varepsilon |\sigma \left(t\right)-s|$$

for all s∈U. If *u* is Δ–differentiable at every $t\in T^{\kappa }$, then *u* is said to be Δ–differentiable. Moreover, if *u* is Δ–differentiable at *t*, then *u* is continuous at *t*, and so, if *u* is Δ–differentiable then u∈C(T). Denote by $C_{\mathrm{rd}}^{1}\left(T\right)$ the set of functions u∈C(T), which are Δ−differentiable on $T^{\kappa }$, and their Δ−derivatives are rd–continuous on $T^{\kappa }$ with the norm

$$\begin{array}{c} {∥u∥}_{T}^{1}={∥u∥}_{T}+{∥u^{\Delta }∥}_{T^{\kappa }}. \end{array}$$

The space $C_{\mathrm{rd}}^{1}\left(T\right)$ is a Banach space.

We say that $v:T^{\kappa }\to R$ is Δ−weak derivative of *u* if

$$\begin{array}{c} \int_{T}\left(u\cdot \varphi^{\Delta }\right)\left(s\right)\Delta s=-\int_{T}\left(v\cdot \varphi^{\sigma }\right)\left(s\right)\Delta s \end{array}$$

for every $\varphi \in C_{0,\mathrm{rd}}^{1}\left(T\right)$, where $C_{0,\mathrm{rd}}^{1}\left(T\right)=\left\{u\in C_{\mathrm{rd}}^{1}\left(T\right):u\left(a\right)=u\left(b\right)=0\right\}$. We denote $v=\Delta^{w}u$.

Given a function u:T→R, we consider an auxiliary function which extends *u* to the real interval [a,b], $\bar{u}:\left[a,b\right]\to R$ defined as

(7) $$\begin{array}{c} \bar{u}\left(t\right)=\left\{\begin{array}{cc} u(t) & \mathrm{for}t\in T\\ u\left(s\right)+\frac{u(\sigma (s))-u(s)}{\mu (s)}\left(t-s\right) & \mathrm{for}t\in \left(s,\sigma \left(s\right)\right),\;s\in R_{T} \end{array}\right.. \end{array}$$

**Lemma** **1.** *The following statements are equivalent*

*(i)*  *u maps every Δ−null subset of T into a null set;*
*(ii)*  *$\bar{u}$ maps every null subset of [a,b] into a null set.*

**Proof.**  From [17], we know that conditions (i) and (ii) are equivalent in the case when the point *b* defined in (2) does not contain Δ−null subsets of time scale T. Therefore, since we adopted the approach to the Δ−measure from [11], it is sufficient to show that $\mu_{L}\left(u\left(\left\{b\right\}\right)\right)=\mu_{L}\left(\bar{u}\left(\left\{b\right\}\right)\right)$. Indeed, we obtain $\bar{u}\left(b\right)=u\left(b\right)$. Consequently, u({b}) is a null set if and only if $\bar{u}\left(\left\{b\right\}\right)$ is a null set. □

A partition of T is a finite ordered subset $P=\{x_{0},x_{1},\ldots ,x_{n-1},x_{n}\}\subset T$, where $a=x_{0}<x_{1}<\ldots <x_{n-1}<x_{n}=b$, with a,b as in (2). For u:T→R and a partition $P=\{x_{0},x_{1},\ldots ,x_{n-1},x_{n}\}\subset T$, we define

$$\begin{array}{c} V\left(P,u\right)=\sum_{k=1}^{n}\left|u\left(x_{k}\right)-u\left(x_{k-1}\right)\right|. \end{array}$$

The total variation of *u* on T is given by $V_{a}^{b}=\sup\left\{V\left(P,u\right):P\;\mathrm{partition}\;\mathrm{of}\;T\right\}$, $V_{a}^{b}\in \left[0,\infty \right]$. If $V_{a}^{b}\in R$, we say that *u* is a function of bounded variation on T.

A direct consequence of the definition of $\bar{u}$ is the following result.

**Proposition** **1.** *Ref.* [17]*. Let u:T→R and $\bar{u}:\left[a,b\right]\to R$ be the extension of u to [a,b] defined in (7). Then, u is of bounded variation on T if and only if $\bar{u}$ is of bounded variation on [a,b].*

A function u:T→R is said to be absolutely continuous if for every ε>0 there exists a δ>0 such that if ${\left\{\left[a_{k},b_{k}\right]\cap T\right\}}_{k=1}^{n}$, with $a_{k},b_{k}\in T$, is a finite pairwise disjoint family of subintervals of T satisfying $\sum_{k=1}^{n}\left(b_{k}-a_{k}\right)<\delta$, then $\sum_{k=1}^{n}\left|u\left(b_{k}\right)-u\left(a_{k}\right)\right|<\varepsilon$. We denote by AC(T) the set of all absolutely continous functions over T.

The following results establish a criterion for absolute continuity on the time scale T.

**Proposition** **2.** *Ref.* [17]*. A function u:T→R is absolutely continuous on T if the following conditions hold true*

*(i)*  *u is continuous and of bounded variation on T;*
*(ii)*  *u maps every* Δ*–null subset of T into a null set.*

**Proposition** **3.** *Ref.* [17]*. Function u:T→R is absolutely continuous on T if and only if the extension function $\bar{u}$ defined in (7) is absolutely continuous on [a,b].*

Now, we can formulate the Fundamental Theorem of Calculus.

**Proposition** **4.** *Ref.* [17]*. A function u:T→R is absolutely continuous on T if and only if the following conditions are satisfied*

*(i)*  *u is* Δ*–differentiable* Δ*–a.e. on T and $u^{\Delta }\in L^{1}\left(T\right)$;*
*(ii)*  *The equality $u\left(t\right)=u\left(a\right)+\int_{a}^{t}u^{\Delta }\left(s\right)\Delta s$ holds for every t∈T.*

We call $f:T\times R^{n}\to R^{n}$ a Δ–Carathéodory function if:

(i) y↦f(s,y) is continuous for Δ−a.a. s∈T;
(ii) s↦f(s,y)Δ−measurable for all $y\in R^{n}$.

We call *f* an $L^{1}$–Carathéodory function if *f* is Δ–Carathéodory function and

(iii) for each d>0 there exists a nonnegative $f_{d}\in L^{1}\left(T\right)$ such that ${∥y∥}_{R^{n}}\leq d$ implies $\left|f(s,y)\right|\leq f_{d}\left(s\right)$ for Δ−a.a. s∈T.

Consider a measurable function p:T→(1,∞) and assume that it is bounded, i.e.,

$$\begin{array}{c} 1<p^{-}:=\underset{t\in T}{ess inf}p\left(t\right)\leq \underset{t\in T}{ess sup}p\left(t\right)=:p^{+}<\infty , \end{array}$$

and we write $p\in L_{+}^{\infty }\left(T\right)$.

By M(T), we denote the set of all equivalence classes of real Δ–measurable functions defined on T being equal Δ–a.e. on T. The variable exponent Lebesgue space $L^{p(t)}\left(T\right)$ consists of all measurable functions u∈M(T) for which the $\rho_{p(\cdot )}$–modular

(8) $$\begin{array}{c} \rho_{p(\cdot )}\left(u\right)=\int_{T}{\left|u\left(t\right)\right|}^{p(t)}\Delta t \end{array}$$

is finite, i.e.,

$$\begin{array}{c} L^{p(t)}\left(T\right)=\left\{u\in M\left(T\right)\;:\;\int_{T}{\left|u\left(t\right)\right|}^{p(t)}\Delta t<\infty \right\}. \end{array}$$

The Luxemburg-type norm on this space is defined as

$$\begin{array}{c} {∥u∥}_{L^{p(t)}\left(T\right)}=\inf\left\{\lambda >0:\rho_{p(t)}\left(\frac{u}{\lambda }\right)\leq 1\right\}. \end{array}$$

Equipped with this norm, $L^{p(t)}\left(T\right)$ is separable and reflexive if $p\in L_{+}^{\infty }\left(T\right)$.

For estimates, one can use the following inequalities.

**Proposition** **5.** *Ref.* [15]*. Let $v,w\in L^{p(t)}\left(T\right)$. Then, for Δ−a.a. t∈T,*

*(a)*  *${\left|v\left(t\right)+w\left(t\right)\right|}^{p(t)}\leq 2^{p^{+}-1}\left({\left|v\left(t\right)\right|}^{p(t)}+{\left|w\left(t\right)\right|}^{p(t)}\right)$;*
*(b)*  *${\left|v\left(t\right)-w\left(t\right)\right|}^{p(t)}\leq 2^{p^{+}-1}\left({\left|v\left(t\right)\right|}^{p(t)}+{\left|w\left(t\right)\right|}^{p(t)}\right)$.*

**Proposition** **6.** *Ref.* [15]*. Let $u\in L^{p(t)}\left(T\right),u\neq \theta$. Then,*

*(a)*  *${∥u∥}_{L^{p(t)}\left(T\right)}<1\;\;\left(=1,\;>1\right)\Leftrightarrow \rho \left(u\right)<1\;\;\left(=1,\;>1\right)$;*
*(b)*  *If ${∥u∥}_{L^{p(t)}\left(T\right)}>1$, then ${∥u∥}_{L^{p(t)}\left(T\right)}^{p^{-}}\leq \rho_{p(t)}\left(u\right)\leq {∥u∥}_{L^{p(t)}\left(T\right)}^{p^{+}}$;*
*(c)*  *If ${∥u∥}_{L^{p(t)}\left(T\right)}<1$, then ${∥u∥}_{L^{p(t)}\left(T\right)}^{p^{+}}\leq \rho_{p(t)}\left(u\right)\leq {∥u∥}_{L^{p(t)}\left(T\right)}^{p^{-}}$.*

**Proposition** **7.** *There exist functions $f_{1},f_{2}:\left[0,\infty \right)\to \left[0,\infty \right)$, which are continuous, strongly increasing, $f_{1}\left(0\right)=f_{2}\left(0\right)=0$ and $\lim_{t\to \infty }f_{1}\left(t\right)=\lim_{t\to \infty }f_{2}\left(t\right)=\infty$ such that, for all $u\in L^{p(t)}\left(T\right)$,*

$$\begin{array}{c} f_{1}{(∥u∥}_{L^{p(t)}\left(T\right)}{)∥u∥}_{L^{p(t)}\left(T\right)}\leq \rho_{p(\cdot )}\left(u\right)\leq f_{2}{(∥u∥}_{L^{p(t)}\left(T\right)}{)∥u∥}_{L^{p(t)}\left(T\right)}. \end{array}$$

Note that these inequalities imply the equivalence of convergence in norm and in modular.

**Proposition** **8.** *Ref.* [15]*. Let $u\in L^{p(t)}\left(T\right)$ and $u_{k}\in L^{p(t)}\left(T\right)$ for k∈N. Then,*

$$\lim_{k\to \infty }{∥u_{k}-u∥}_{\rho }=0\;\;\;\;\mathrm{if}\;\mathrm{and}\;\mathrm{only}\;\mathrm{if}\;\;\;\;\lim_{k\to \infty }\rho_{p(\cdot )}\left(\left(u_{k}-u\right)\right)=0.$$

**Lemma** **2.** *Ref.* [15]*. Let ${\left(u_{k}\right)}_{k\in N}\subset L^{p(t)}\left(T\right)$ be a sequence convergent to a certain function $u\in L^{p(t)}\left(T\right)$. Then, there exists a subsequence ${\left(u_{k_{l}}\right)}_{l\in N}\subset L^{p(t)}\left(T\right)$ such that $\lim_{l\to \infty }u_{k_{l}}\left(t\right)=u\left(t\right)$ for Δ−a.a. t∈T and there exists a function $g\in L^{p(t)}\left(T\right)$ such that $|u_{k_{l}}\left(t\right)|\leq g\left(t\right)$ for l∈N and Δ−a.a. t∈T.*

**Proposition** **9.** *Ref.* [15]*. If $p_{1},p_{2}\in L_{+}^{\infty }\left(T\right)$ and $p_{1}\left(t\right)\leq p_{2}\left(t\right)$ for Δ−a.a. t∈T, then the embedding $L^{p_{2}\left(t\right)}\left(T\right)↪L^{p_{1}\left(t\right)}\left(T\right)$ is continuous.*

Let $p,q\in L_{+}^{\infty }\left(T\right)$ and p,q be conjugative on the time scale T, e.g.,

(9) $$\frac{1}{p(t)}+\frac{1}{q(t)}=1$$

for Δ−a.a t∈T. The space $L^{q(t)}\left(T\right)$ is defined as

$$\begin{array}{c} L^{q(t)}\left(T\right)=\left\{u\in M\left(T\right)\;:\;\int_{T}\frac{1}{q(t)}{\left|u\left(t\right)\right|}^{q(t)}\Delta t<\infty \right\}. \end{array}$$

**Proposition** **10.** *Ref.* [15]*. For every $u\in L^{p(t)}\left(T\right)$ and $v\in L^{q(t)}\left(T\right)$, the following Hölder inequality holds:*

(10) $$\begin{array}{c} \int_{T}|u\left(t\right)v\left(t\right)|\Delta t\leq \left(\frac{1}{p^{-}}+\frac{1}{q^{-}}\right){∥u∥}_{L^{p(t)}\left(T\right)}{∥v∥}_{L^{q(t)}\left(T\right)}. \end{array}$$

We define the variable exponent Sobolev space on time scales by

$$\begin{array}{c} W^{1,p(t)}\left(T\right)=\left\{u\in L^{p(t)}\left(T\right)\;:\;\Delta^{w}u\in L^{p(t)}\left(T\right)\right\} \end{array}$$

equipped with the norm

$$\begin{array}{c} {∥u∥}_{W^{1,p(t)}\left(T\right)}={∥u∥}_{L^{p(t)}\left(T\right)}+{∥\Delta^{w}u∥}_{L^{p(t)}\left(T\right)}. \end{array}$$

Then, $\left(W^{1,p(t)}\left(T\right),{∥\;\cdot \;∥}_{W^{1,p(t)}\left(T\right)}\right)$ is separable and reflexive if $p\in L_{+}^{\infty }\left(T\right)$.

We denote by $C_{\mathrm{rd}}^{\infty }\left(T\right)$ (respectively $C^{\infty }\left(T\right)$) the set of continuous functions over T which are of *n* times rd-continuously (respectively continuously) Δ–differentiable on $T^{\kappa }$ for any n∈N. We define $W_{0}^{1,p(t)}\left(T\right)$ as the closure of $C_{0,\mathrm{rd}}^{\infty }\left(T\right)$ in $W^{1,p(t)}\left(T\right)$, where

$$\begin{array}{c} C_{0,\mathrm{rd}}^{\infty }\left(T\right)=\left\{u\in C_{\mathrm{rd}}^{\infty }\left(T\right):u\left(a\right)=u\left(b\right)=0\right\}. \end{array}$$

**Remark** **1.** *In general, $C_{0}^{\infty }\left(\Omega \right)$, $\Omega \subset R^{n}$, may not be dense in $W^{1,p(x)}\left(\Omega \right)$. It is true under some additional assumption upon p (see [7,18]). However, it is known that if $p^{-}\geq n$ then, $C_{0}^{\infty }\left(\Omega \right)$ is dense in $W^{1,p(x)}\left(\Omega \right)$ and*

$$\begin{array}{c} W_{0}^{1,p(x)}\left(\Omega \right)=W^{1,p(x)}\left(\Omega \right)\cap W_{0}^{1,1}\left(\Omega \right). \end{array}$$

*In the classical one-dimensional situation of $W^{1,p(t)}\left(I\right)$ with I=(a,b), each element u has a continuous representative $\bar{u}$ (see (7)) in its equivalence class for equality Δ−a.a., and $W_{0}^{1,p(t)}\left(I\right)$ can be characterized as the set of $u\in W^{1,p(t)}\left(I\right)$ such that $\bar{u}\left(a\right)=0=\bar{u}\left(b\right)$.*

Recall that there exists C>0, such that ${∥u∥}_{L^{p(t)}\left(T\right)}\leq C{∥\Delta^{w}u∥}_{L^{p(t)}\left(T\right)}$ for $u\in W_{0}^{1,p(t)}\left(T\right)$. Consequently, one can consider the space $W_{0}^{1,p(t)}\left(T\right)$ with the following equivalent norm

(11) $$\begin{array}{c} {∥u∥}_{W_{0}^{1,p(t)}\left(T\right)}={∥\Delta^{w}u∥}_{L^{p(t)}\left(T\right)}. \end{array}$$

It is known that the following continuous embeddings hold

(12) $$\begin{array}{c} C\left(T\right)↪C_{\mathrm{rd}}\left(T\right)↪L^{p^{+}}\left(T\right)↪L^{p(t)}\left(T\right)↪L^{p^{-}}\left(T\right) \end{array}$$

and

$$\begin{array}{c} W^{1,p^{+}}\left(T\right)↪W^{1,p(t)}\left(T\right)↪W^{1,p^{-}}\left(T\right). \end{array}$$

Moreover, we recall that the following embeddings

(13) $$\begin{array}{c} W^{1,p(t)}\left(T\right)↪C\left(T\right),\;\;\mathrm{and}\;\;W^{1,p(t)}\left(T\right)↪L^{p^{+}}\left(T\right) \end{array}$$

are compact.

Since any element of $W^{1,p^{-}}\left(T\right)$ is absolutely continuous (see [14]), we know that the same holds for any $u\in W^{1,p(t)}\left(T\right)$, which implies that any element of $W^{1,p(t)}\left(T\right)$ is Δ–differentiable Δ–a.e. on T. If $u\in W_{0}^{1,p(t)}\left(T\right)$, then *u* is continuous, which implies that $u^{\sigma }\in C_{\mathrm{rd}}\left(T\right)$ and (6) holds. Consequently, by (12) and (13), there are $A,C,C_{1}>0$ such that

(14) $$\begin{array}{c} {∥u^{\sigma }∥}_{L^{p(t)}\left(T\right)}\leq C{∥u^{\sigma }∥}_{L^{p^{+}}\left(T\right)}\leq C_{1}{∥u^{\sigma }∥}_{T}\leq C_{1}{∥u∥}_{T}\leq C_{1}A{∥u∥}_{X}. \end{array}$$

## 3. Du Bois−Reymond Type Lemma

In this section, we will prove a du Bois–Reymond type lemma for nondifferentiable functions.

By (9), we estimate

(15) $$\begin{array}{c} \int_{T}\frac{1}{q(t)}{\left({\left|u\left(t\right)\right|}^{p(t)-1}\right)}^{q(t)}\Delta t\leq \frac{1}{q^{-}}\int_{T}{\left|u\left(t\right)\right|}^{p(t)}\Delta t<\infty \end{array}$$

for any $u\in L^{p(t)}\left(T\right)$. Consequently,

(16) $$\begin{array}{c} {\left|u\left(t\right)\right|}^{p(t)-2}u\left(t\right)\in L^{q(t)}\left(T\right) \end{array}$$

for $u\in L^{p(t)}\left(T\right)$, where *q* is the function given in (9). By (10) and (15), for any $u,v\in L^{p(t)}\left(T\right)$,

$$\begin{array}{c} \int_{T}{\left|u\left(t\right)\right|}^{p(t)-2}u\left(t\right)v\left(t\right)\Delta t \end{array}$$

is well defined.

**Lemma** **3.** *If $h\in L^{q(t)}\left(T\right)$ and*

$$\begin{array}{c} \int_{T}h\left(t\right)v^{\Delta }\left(t\right)\Delta t=0 \end{array}$$

*for every $v\in W_{0}^{1,p(t)}\left(T\right)$, then*

h(t)=const.

*for Δ−a.a. t∈T.*

**Proof.**  Let us define

$$v\left(t\right)=\int_{a}^{t}{\left|h(s)-c\right|}^{q(s)-2}\left(h(s)-c\right)\Delta s$$

for t∈T, where c∈R is such that v(b)=0. Then, v(a)=0. Moreover,

(17) $$\begin{array}{c} v^{\Delta }\left(t\right)={\left|h(t)-c\right|}^{q(t)-2}\left(h(t)-c\right) \end{array}$$

for Δ–a.a. t∈T and $v^{\Delta }\in L^{p(t)}\left(T\right)$. By (17), we have

$$\begin{array}{ccc} \int_{T}{\left|h(t)-c\right|}^{q(t)}\Delta t & = & \int_{T}\left(h(t)-c\right){\left|h(t)-c\right|}^{q(t)-2}\left(h(t)-c\right)\Delta \;t\\ & = & \int_{T}\left(h(t)-c\right)v^{\Delta }\left(t\right)\Delta \;t\\ & = & \int_{T}h\left(t\right)v^{\Delta }\left(t\right)\Delta t-c\int_{T}v^{\Delta }\Delta t=0. \end{array}$$

Since $\rho_{q(\cdot )}$ is a modular, we have h(t)=c for Δ−a.a. t∈T and the lemma follows. □

The following lemma plays a key role in the next section.

**Lemma** **4.** *Let $h_{1}\in L^{1}\left(T\right),h_{2}\in L^{p(t)}\left(T\right)$ and*

(18) $$\begin{array}{c} \int_{T}\left(h_{1}\left(t\right)v^{\sigma }\left(t\right)+{\left|h_{2}\left(t\right)\right|}^{p(t)-2}h_{2}\left(t\right)v^{\Delta }\left(t\right)\right)\Delta t=0 \end{array}$$

*for every $v\in W_{0}^{1,p(t)}\left(T\right)$. Then,*

$$h_{1}\left(t\right)=\frac{\Delta }{\Delta t}\left({\left|h_{2}\left(t\right)\right|}^{p(t)-2}h_{2}\left(t\right)\right)$$

*for Δ−a.a. t∈T.*

**Proof.**  Let

$$H\left(t\right)=\int_{a}^{t}h_{1}\left(s\right)\Delta s$$

for t∈T. Integrating by parts and using the boundary conditions,

(19) $$\begin{array}{ccc} \int_{T}h_{1}\left(t\right)v^{\sigma }\left(t\right)\Delta t & = & \int_{T}H^{\Delta }\left(t\right)v^{\sigma }\left(t\right)\Delta t=H\left(t\right)v\left(t\right){|}_{a}^{b}-\int_{T}v^{\Delta }\left(t\right)H\left(t\right)\Delta t\\ & = & -\int_{T}v^{\Delta }\left(t\right)H\left(t\right)\Delta t \end{array}$$

for every $v\in W_{0}^{1,p(t)}\left(T\right)$. By (18) and (19), we obtain

$$\begin{array}{ccc} 0 & = & \int_{T}\left(h_{1}\left(t\right)v^{\sigma }\left(t\right)+{\left|h_{2}\left(t\right)\right|}^{p(t)-2}h_{2}\left(t\right)v^{\Delta }\left(t\right)\right)\Delta \;t\\ & = & -\int_{T}v^{\Delta }\left(t\right)H\left(t\right)\Delta t+\int_{T}{\left|h_{2}\left(t\right)\right|}^{p(t)-2}h_{2}\left(t\right)v^{\Delta }\left(t\right)\Delta \;t\\ & = & \int_{T}v^{\Delta }\left(t\right)\left(|h_{2}{\left(t\right)|}^{p(t)-2}h_{2}\left(t\right)-H\left(t\right)\right)\Delta t \end{array}$$

for every $v\in W_{0}^{1,p(t)}\left(T\right)$. Now, combining (16) with (12) and Lemma 3, we obtain that there exists c∈R, such that

$$|h_{2}{\left(t\right)|}^{p(t)-2}h_{2}\left(t\right)=H\left(t\right)+c$$

and

$$\frac{\Delta }{\Delta t}\left(|h_{2}{\left(t\right)|}^{p(t)-2}h_{2}\left(t\right)\right)=\frac{\Delta }{\Delta t}\left(H(t)+c\right)=h_{1}\left(t\right)$$

for Δ−a.a. t∈T. □

**Corollary** **1.** *If $h\in L^{1}\left(T\right)$ and*

$$\int_{T}h\left(t\right)v^{\sigma }\left(t\right)\Delta t=0$$

*for every $v\in W_{0}^{1,p(t)}$, then h(t)=0 for Δ−a.a. t∈T.*

**Proof.**  It suffices to take $h_{2}\left(t\right)=0$ for Δ−a.a. t∈T in Lemma 4. □

## 4. The p(t)–Laplacian Dirichlet Problem

Let $X:=W_{0}^{1,p(t)}\left(T\right)$. The following assumptions upon *f* and *p* are made:

(P) $p\in L_{+}^{\infty }\left(T\right)$;
(F) f:T×R→R is a $L^{1}-$Carathéodory function over T×R.

Let us consider the following problem:

(20) $$\left\{\begin{array}{c} -\Delta_{p(t)}u\left(t\right):=-\frac{\Delta }{\Delta t}\left(|\Delta^{w}{u\left(t\right)|}^{p(t)-2}\Delta^{w}u\left(t\right)\right)=f\left(t,u^{\sigma }\left(t\right)\right),\;t\in T\\ u(a)=u(b)=0 \end{array}\right.,$$

where u∈X, *a* and *b* are defined in (2) and σ is a forward jump operator given in (3).

We say that u∈X is a weak solution to (20) if

(21) $$\int_{T}{\left|\Delta^{w}u\left(t\right)\right|}^{p(t)-2}\Delta^{w}u\left(t\right)\Delta^{w}v\left(t\right)\Delta t=\int_{T}f\left(t,u^{\sigma }\left(t\right)\right)v^{\sigma }\left(t\right)\Delta t$$

for every v∈X.

We define the functional φ:X→R by

(22) $$\varphi \left(u\right)=\int_{T}\frac{1}{p(t)}{\left|\Delta^{w}u\left(t\right)\right|}^{p(t)}\Delta t-\int_{T}F\left(t,u^{\sigma }\left(t\right)\right)\Delta t,$$

where

(23) $$F\left(t,x\right)=\int_{0}^{x}f\left(t,s\right)ds$$

for Δ−a.a. t∈T and x∈R. Moreover, let us denote

(24) $$\varphi_{1}\left(u\right)=\int_{T}\frac{1}{p(t)}{\left|\Delta^{w}u\left(t\right)\right|}^{p(t)}\Delta t$$

and

(25) $$\varphi_{2}\left(u\right)=\int_{T}F\left(t,u^{\sigma }\left(t\right)\right)\Delta t$$

for u∈X.

Observe that if *f* satisfies Assumption (F), then also *F* is an $L^{1}$–Carathéodory function over T×R and thus, $t\mapsto F(t,u^{\sigma }\left(t\right))$ belongs to $L^{1}\left(T\right)$. Consequently, $\varphi_{2}$ is well defined, which implies that φ is well defined.

**Lemma** **5.** *The functional $\varphi_{1}$ defined in (24) is continuously differentiable on X at any u∈X and*

$$\varphi_{1}^{\prime }\left(u\right)\left(v\right)=\int_{T}{\left|\Delta^{w}u\left(t\right)\right|}^{p(t)-2}\Delta^{w}u\left(t\right)\Delta^{w}v\left(t\right)\Delta t$$

*for all v∈X.*

**Proof.**  Let us define

$$\begin{array}{ccc} \tilde{\varphi_{1}}\left(\lambda ,t\right) & = & \frac{1}{p(t)}{\left|\Delta^{w}u\left(t\right)+\lambda \Delta^{w}v\left(t\right)\right|}^{p(t)}\\ & = & \frac{1}{p(t)}{\left[{\left(\Delta^{w}u\left(t\right)+\lambda \Delta^{w}v\left(t\right)\right)}^{2}\right]}^{\frac{p(t)}{2}} \end{array}$$

and

$$\Psi_{1}\left(\lambda \right)=\int_{T}\tilde{\varphi_{1}}\left(\lambda ,t\right)\Delta t=\varphi_{1}\left(u+\lambda v\right),$$

where u,v∈X are fixed, t∈T and λ∈[−1,1]. Consequently,

$$\begin{array}{c} \varphi_{1}^{\prime }\left(u\right)\left(v\right)=\Psi_{1}^{\prime }\left(0\right)=\int_{T}\tilde{\varphi_{1\lambda }^{\prime }}{\left(\lambda ,t\right)|}_{\lambda =0}\Delta t=\int_{T}{\left|\Delta^{w}u\left(t\right)\right|}^{p(t)-2}\Delta^{w}u\left(t\right)\Delta^{w}v\left(t\right)\Delta t. \end{array}$$

Let us define

$$\begin{array}{c} u_{1}\left(t\right)={\left|\Delta^{w}u\left(t\right)\right|}^{p(t)-1} \end{array}$$

for t∈T. By (16), $u_{1}\in L^{q(t)}\left(T\right)$. By Hölder inequality (10) and (11), we obtain

$$\begin{array}{c} |\varphi_{1}^{\prime }\left(u\right)\left(v\right)|\leq \left(\frac{1}{p^{-}}+\frac{1}{q^{-}}\right)∥u_{1}{∥}_{L^{q(t)}\left(T\right)}{∥v∥}_{X}. \end{array}$$

Consequently, $\varphi_{1}^{\prime }\left(u\right)\in X^{*}$ and functional $\varphi_{1}$ is Gâteaux differentiable over *X*.

We shall show that the derivative is continuous. Consider $\xi_{p(\cdot )}:L^{p(t)}\left(T\right)\to L^{q(t)}\left(T\right)$ given by

$$\begin{array}{c} \xi_{p(t)}\left(u\right)={\left|u\right|}^{p(t)-2}u \end{array}$$

for $u\in L^{p(t)}\left(T\right)$. By (15), $\xi_{p(\cdot )}$ is well defined.

Let $u_{n}\to u$ in $L^{p(t)}\left(T\right)$ and $\left(v_{n}\right)$ be a subsequence of $\left(u_{n}\right)$. Let $\left(v_{n_{l}}\right)$ and *g* be given as in Lemma 2. Then, from Lemma 2 and Proposition 5, one has

$$\begin{array}{ccc} {\left|\xi_{p(t)}\left(v_{n_{l}}\right)-\xi_{p(t)}\left(u\right)\right|}^{q(t)} & \leq & 2^{q^{+}-1}\left\{{\left(|v_{n_{l}}{\left(t\right)|}^{p(t)-1}\right)}^{q(t)}+{\left({\left|u\left(t\right)\right|}^{p(t)-1}\right)}^{q(t)}\right\}\\ & \leq & 2^{q^{+}-1}\left\{|v_{n_{l}}{\left(t\right)|}^{p(t)}+{\left|u\left(t\right)\right|}^{p(t)}\right\}\\ & \leq & 2^{q^{+}}{\left(g\left(t\right)\right)}^{p(t)}. \end{array}$$

Since $v_{n_{l}}\left(t\right)\to u\left(t\right)$ for Δ−a.a. t∈T, it follows from Lebesgue Dominated Convergence Theorem that

$$\begin{array}{c} \int_{T}{\left|\xi_{p(t)}\left(v_{n_{l}}\right)-\xi_{p(t)}\left(u\right)\right|}^{q(t)}\Delta t\to 0,\;\;\mathrm{as}\;l\to \infty , \end{array}$$

but then, since any subsequence $\left(\xi_{p(t)}\left(v_{n}\right)\right)$ has a subsequence $\left(\xi_{p(t)}\left(v_{n_{l}}\right)\right)$ convergent to the same limit,

$$\begin{array}{c} \int_{T}{\left|\xi_{p(t)}\left(u_{n}\right)-\xi_{p(t)}\left(u\right)\right|}^{q(t)}\Delta t\to 0,\;\;\mathrm{as}\;n\to \infty . \end{array}$$

Consequently, using Proposition 8 and Hölder inequality (10),

$$\begin{array}{c} \left|\varphi_{1}^{\prime }\left(u_{n}\right)\left(v\right)-\varphi_{1}^{\prime }\left(u\right)\left(v\right)\right|\leq \left(\frac{1}{p^{-}}+\frac{1}{q^{-}}\right){∥\xi_{p(t)}\left(\Delta^{w}u_{n}\right)-\xi_{p(t)}\left(\Delta^{w}u\right)∥}_{L^{q(t)}}{∥v∥}_{X} \end{array}$$

and

$$\begin{array}{c} \lim_{n\to \infty }{∥\varphi_{1}^{\prime }\left(u_{n}\right)-\varphi_{1}^{\prime }\left(u\right)∥}_{X^{*}}=0. \end{array}$$

□

**Lemma** **6.** *The functional $\varphi_{2}$ defined in (25) is continuously differentiable on X at any u∈X and*

$$\varphi_{2}^{\prime }\left(u\right)\left(v\right)=\int_{T}f\left(t,u^{\sigma }\left(t\right)\right)v^{\sigma }\left(t\right)\Delta t$$

*for all v∈X.*

**Proof.**  Let us define

$$\tilde{\varphi_{2}}\left(\lambda ,t\right)=F\left(t,u^{\sigma }\left(t\right)+\lambda v^{\sigma }\left(t\right)\right)$$

and

$$\Psi_{2}\left(\lambda \right)=\int_{T}\tilde{\varphi_{2}}\left(\lambda ,t\right)\Delta t=\varphi_{2}\left(u+\lambda v\right),$$

where u,v∈X are fixed, t∈T and λ∈[−1,1]. Thus, we get

$$\begin{array}{c} \varphi_{2}^{\prime }\left(u\right)\left(v\right)=\Psi_{2}^{\prime }\left(0\right)=\int_{T}\tilde{\varphi_{2\lambda }^{\prime }}{\left(\lambda ,t\right)|}_{\lambda =0}\Delta t=\int_{T}f\left(t,u^{\sigma }\left(t\right)\right)v^{\sigma }\left(t\right)\Delta t. \end{array}$$

By (14) and since $t\mapsto f(t,u^{\sigma }\left(t\right))$ belongs to $L^{1}\left(T\right)$, we have

$$\begin{array}{c} |\varphi_{2}^{\prime }{\left(u\right)\left(v\right)|\leq ∥v∥}_{T}\int_{T}|f\left(t,u^{\sigma }\left(t\right)\right){|\Delta t\leq A∥v∥}_{X}\int_{T}\left|f\left(t,u^{\sigma }\left(t\right)\right)\right|\Delta t<\infty . \end{array}$$

Therefore, $\varphi_{2}^{\prime }\left(u\right)\in X^{*}$ and functional $\varphi_{2}$ is Gâteaux differentiable over *X*.

If $u_{n}\to u$ in *X*, then, by (14) $,u_{n}\to u$ in C(T) and there exists d>0 such that $|u_{n}\left(t\right)|\leq d$ for n∈N, which implies that $|u_{n}^{\sigma }\left(t\right)|\leq d$ for n∈N and Δ−a.a. t∈T. Since *f* is $L^{1}-$Carathéodory function, there is $f_{d}\in L^{1}\left(T\right)$ such that, for n∈N and for Δ−a.a. t∈T, we have $|f\left(t,u_{n}^{\sigma }\left(t\right)\right)|\leq f_{d}\left(t\right)$.

Let $u_{n}\to u$ in *X*. Then, from (14), $u_{n}^{\sigma }\to u^{\sigma }$ in $L^{p(t)}\left(T\right)$. Now, as in the second part of the proof of Lemma 5, using Lemma 2, one can show that $f\left(t,u_{n}^{\sigma }\left(t\right)\right)\to f\left(t,u^{\sigma }\left(t\right)\right)$ for Δ–a.a. t∈T, as n→∞. Applying the Lebesgue Dominated Convergence Theorem, $\varphi_{2}$ is continuously differentiable. □

**Remark** **2.** *From Lemmas 5 and 6, a critical point of functional φ defined in (22) is also a weak solution to (20). Now, taking*

$$\begin{array}{c} h_{1}\left(t\right):=-f\left(t,u^{\sigma }\left(t\right)\right)\;\mathrm{and}\;h_{2}\left(t\right):=\Delta^{w}u\left(t\right)\;\mathrm{for}\;t\in T\; \end{array}$$

*in Lemma 4, we obtain that a possible solution to (21) is a solution to problem (20).*

*Moreover, from Lemma 4, the function*

$$\begin{array}{c} t\mapsto |\Delta^{w}{u\left(t\right)|}^{p(t)-2}\Delta^{w}u\left(t\right) \end{array}$$

*is absolutely continuous on T. Consequently, a weak solution to problem (20) is a classical solution.*

We now provide some properties of the operator $\varphi_{1}$ that will be needed in next Sections.

It is easy to verify that the following holds: if $p_{0}\in \left(1,\infty \right)$, then

(26) $$\begin{array}{c} \langle {\left|x\right|}^{p_{0}-2}x-{\left|y\right|}^{p_{0}-2}y,x-y\rangle \geq \left|{\left|x\right|}^{p_{0}-1}-{\left|y\right|}^{p_{0}-1}\right|\left||x\right|-\left|y|\right| \end{array}$$

for all $x,y\in R^{n}$.

**Lemma** **7.** The mapping $\varphi_{1}^{\prime }:X\to X^{*}$ is coercive and strictly monotone.

**Proof.**  Observe that, from (26), $\varphi_{1}^{\prime }$ is strictly monotone. Moreover, by Proposition 6, one has

$$\begin{array}{c} \lim_{{∥u∥}_{X}\to \infty }\frac{\varphi_{1}^{\prime }\left(u\right)\left(u\right)}{{∥u∥}_{X}}=\lim_{{∥u∥}_{X}\to \infty }\frac{\rho_{p(\cdot )}\left(\Delta^{w}u\right)}{{∥u∥}_{X}}\geq \lim_{{∥u∥}_{X}\to \infty }\frac{{∥\Delta^{w}u∥}_{L^{p(t)}\left(T\right)}^{p^{-}}}{{∥u∥}_{X}}=\infty . \end{array}$$

Consequently, $\varphi_{1}^{\prime }$ is coercive. □

**Lemma** **8.** The operator $\varphi_{1}^{\prime }:X\to X^{*}$ is demicontinuous.

**Proof.**  Since $\varphi_{1}^{\prime }$ is continuous, one can easily see that $\varphi_{1}^{\prime }$ is hemicontinuous, i.e., for all u,v,w∈X, the mapping $h\to \varphi_{1}^{\prime }\left(u+hv\right)\left(w\right)$ is continuous on [0,1]. Now, the statement follows from the fact that for a monotone operator, demicontinuity and hemicontinuity are equivalent. □

**Lemma** **9.** The mapping $\varphi_{1}^{\prime }:X\to X^{*}$ is bounded.

**Proof.**  We need to show that $\varphi_{1}^{\prime }$ maps bounded sets in *X* into bounded sets in $X^{*}$. Let u,v∈X and let us define

$$\begin{array}{c} u_{1}\left(t\right)={\left|\Delta^{w}u\left(t\right)\right|}^{p(t)-1} \end{array}$$

for t∈T. By (16), we have $\rho_{q(\cdot )}\left(u_{1}\right)=\rho_{p(\cdot )}\left(\Delta^{w}u\right)<\infty$. Consequently, $u_{1}\in L^{q(t)}\left(T\right)$. By (14) and Hölder inequality (10), we estimate

(27) $$\begin{array}{c} \varphi_{1}^{\prime }\left(u\right)\left(v\right)\leq \left(\frac{1}{p^{-}}+\frac{1}{q^{-}}\right){∥v∥}_{X}{∥u_{1}∥}_{L^{q(t)}\left(T\right)}. \end{array}$$

By Lemma 7, we know that there exists function $f_{1}:\left[0,\infty \right)\to \left[0,\infty \right)$, such that

(28) $$\begin{array}{c} f_{1}\left(∥u_{1}{∥}_{L^{q(t)}\left(T\right)}\right){∥u_{1}∥}_{L^{q(t)}\left(T\right)}\leq \rho_{q(\cdot )}\left(u_{1}\right). \end{array}$$

By (27) and (28), we obtain

(29) $$\begin{array}{ccc} \varphi_{1}^{\prime }\left(u\right)\left(v\right) & \leq & K_{1}\left(\frac{1}{p^{-}}+\frac{1}{q^{-}}\right){∥v∥}_{X}\rho_{q(\cdot )}\left(u_{1}\right)\\ & = & K_{1}\left(\frac{1}{p^{-}}+\frac{1}{q^{-}}\right){∥v∥}_{X}\rho_{p(\cdot )}\left(\Delta^{w}u\right), \end{array}$$

where $K_{1}={\left(f_{1}\left(∥u_{1}{∥}_{L^{q(t)}\left(T\right)}\right)\right)}^{-1}$. From Lemma 7, there is $f_{2}:\left[0,\infty \right)\to \left[0,\infty \right)$, such that

(30) $$\begin{array}{c} \rho_{p(\cdot )}\left(\Delta^{w}u\right)\leq f_{2}\left(∥\Delta^{w}{u∥}_{L^{p(t)}\left(T\right)}\right)∥\Delta^{w}{u∥}_{L^{p(t)}\left(T\right)}=f_{2}{(∥u∥}_{X}{)∥u∥}_{X}. \end{array}$$

By (29) and (30), we estimate

$$\begin{array}{ccc} \varphi_{1}^{\prime }\left(u\right)\left(v\right) & \leq & K_{2}\left(\frac{1}{p^{-}}+\frac{1}{q^{-}}\right){∥v∥}_{X}{∥u∥}_{X}, \end{array}$$

where $K_{2}=K_{1}f_{2}{(∥u∥}_{X})$ and consequently

$$\begin{array}{c} ∥\varphi_{1}^{\prime }{\left(u\right)∥}_{X^{*}}=\underset{{∥v∥}_{X}=1}{\sup}\left|\left(\varphi_{1}^{\prime }u\right)\left(v\right)\right|\leq K_{2}\left(\frac{1}{p^{-}}+\frac{1}{q^{-}}\right){∥u∥}_{X}, \end{array}$$

which implies that the operator $\varphi_{1}^{\prime }$ is bounded. □

**Lemma** **10.** *If $u_{n}⇀u$ in X and*

(31) $$\begin{array}{c} \lim_{n\to \infty }\left(\varphi_{1}^{\prime }\left(u_{n}\right)-\varphi_{1}^{\prime }\left(u\right)\right)\left(u_{n}-u\right)=0, \end{array}$$

*then $u_{n}\to u$ in X.*

**Proof.**  Assume that $u_{n}⇀u$ in *X* and (31) is satisfied. Let

$$\begin{array}{c} T_{1}=\left\{t\in T:1<p(t)<2\right\} \end{array}$$

and

$$\begin{array}{c} T_{2}=\left\{t\in T:p(t)\geq 2\right\}. \end{array}$$

Since the following inequalities hold

(32) $$\begin{array}{ccc} \; & \; & \left({\left|\alpha \right|}^{p_{0}-2}\alpha -{\left|\beta \right|}^{p_{0}-2}\beta \right)\left(\alpha -\beta \right)\geq 2^{2-p_{0}}{\left|\alpha -\beta \right|}^{p_{0}},\;\;p_{0}\geq \;2\\ \; & \; & \left({\left|\alpha \right|}^{p_{0}-2}\alpha -{\left|\beta \right|}^{p_{0}-2}\beta \right)\left(\alpha -\beta \right)\geq \left(p_{0}-1\right){\left|\alpha -\beta \right|}^{2}{\left(1+{\left|\alpha \right|}^{2}+{\left|\beta \right|}^{2}\right)}^{\frac{p_{0}-2}{2}},\\ \; & \; & 1<p_{0}<2 \end{array}$$

for any α,β∈R (see [19]), we get

$$\begin{array}{ccc} & & \left(\varphi_{1}^{\prime }\left(u_{n}\right)-\varphi_{1}^{\prime }\left(u\right)\right)\left(u_{n}-u\right)\\ & = & \int_{T}\left(|\Delta^{w}u_{n}{\left(t\right)|}^{p(t)-2}\Delta^{w}u_{n}\left(t\right)-{\left|\Delta^{w}u\left(t\right)\right|}^{p(t)-2}\Delta^{w}u\left(t\right)\right)\left(\Delta^{w}u_{n}\left(t\right)-\Delta^{w}u\left(t\right)\right)\Delta \;t\\ & = & \int_{T_{1}}\left(|\Delta^{w}u_{n}{\left(t\right)|}^{p(t)-2}\Delta^{w}u_{n}\left(t\right)-{\left|\Delta^{w}u\left(t\right)\right|}^{p(t)-2}\Delta^{w}u\left(t\right)\right)\left(\Delta^{w}u_{n}\left(t\right)-\Delta^{w}u\left(t\right)\right)\Delta \;t\\ & & +\int_{T_{2}}\left(|\Delta^{w}u_{n}{\left(t\right)|}^{p(t)-2}\Delta^{w}u_{n}\left(t\right)-{\left|\Delta^{w}u\left(t\right)\right|}^{p(t)-2}\Delta^{w}u\left(t\right)\right)\left(\Delta^{w}u_{n}\left(t\right)-\Delta^{w}u\left(t\right)\right)\Delta \;t\\ & = & I_{1}+I_{2}. \end{array}$$

From (32), for Δ–a.a. t∈T, we obtain

$$\begin{array}{c} I_{1}\geq \left(p^{-}-1\right)\int_{T}\frac{{\left|\Delta^{w}u\left(t\right)-\Delta^{w}w\left(t\right)\right|}^{2}}{{\left(1+|\Delta^{w}{u\left(t\right)|}^{2}+{\left|\Delta^{w}w\left(t\right)\right|}^{2}\right)}^{\frac{2}{p(t)-2}}}\;\Delta t\geq 0 \end{array}$$

and

$$\begin{array}{c} I_{2}\geq 2^{2-p^{+}}\int_{T}{\left|\Delta^{w}u_{n}\left(t\right)-\Delta^{w}u\left(t\right)\right|}^{p(t)}\Delta t\geq 0. \end{array}$$

Consequently, from (31), $\Delta^{w}u_{n}$ converges in measure to $\Delta^{w}u$. Let us consider a subsequence of $\left(\Delta^{w}u_{n}\right)$ and denote it also by $\left(\Delta^{w}u_{n}\right)$, $\Delta^{w}u_{n}\to \Delta^{w}u$ for Δ–a.a. t∈T. By the Fatou Lemma,

(33) $$\begin{array}{c} \underset{n\to \infty }{lim inf}\int_{T}\frac{1}{p(t)}|\Delta^{w}u_{n}{\left(t\right)|}^{p(t)}\Delta t\geq \int_{T}\frac{1}{p(t)}{\left|\Delta^{w}u\left(t\right)\right|}^{p(t)}\Delta t. \end{array}$$

Since $u_{n}⇀u$ and from (31), we have

(34) $$\begin{array}{c} \lim_{n\to \infty }\left(\varphi_{1}^{\prime }\left(u_{n}\right)-\varphi_{1}^{\prime }\left(u\right)\right)\left(u_{n}-u\right)=\left(\varphi_{1}^{\prime }\left(u_{n}\right)\right)\left(u_{n}-u\right)=0. \end{array}$$

Moreover,

$$\begin{array}{c} \varphi_{1}^{\prime }\left(u_{n}\right)\left(u_{n}-u\right)\geq \int_{T}|\Delta^{w}u_{n}\left(t\right){|}^{p(t)}\Delta t-\int_{T}|\Delta^{w}u_{n}\left(t\right){|}^{p(t)-1}\left|\Delta^{w}u\left(t\right)\right|\Delta t. \end{array}$$

Now, from the Young inequality, one has

$$\begin{array}{c} |\Delta^{w}u_{n}{\left(t\right)|}^{p(t)-1}|\Delta^{w}u\left(t\right)|\leq \frac{p(t)-1}{p(t)}|\Delta^{w}u_{n}{\left(t\right)|}^{p(t)}+\frac{1}{p(t)}{\left|\Delta^{w}u\left(t\right)\right|}^{p(t)}. \end{array}$$

Therefore,

(35) $$\begin{array}{c} \varphi_{1}^{\prime }\left(u_{n}\right)\left(u_{n}-u\right)\geq \int_{T}\frac{1}{p(t)}|\Delta^{w}u_{n}{|}^{p(t)}\Delta t-\int_{T}\frac{1}{p(t)}{\left|\Delta^{w}u\right|}^{p(t)}\Delta t. \end{array}$$

From (31), (33)–(35), we obtain

$$\begin{array}{c} \lim_{n\to \infty }\int_{T}\frac{1}{p(t)}|\Delta^{w}u_{n}{\left(t\right)|}^{p(t)}\Delta t=\int_{T}\frac{1}{p(t)}{\left|\Delta^{w}u\left(t\right)\right|}^{p(t)}\Delta t. \end{array}$$

Consequently, functions $\frac{1}{p(t)}{\left|\Delta^{w}u_{n}\left(t\right)\right|}^{p(t)}$ have equi-absolutely continuous integrals (see ([20], Theorem 3, p. 153)). Moreover, from Proposition 5,

$$\begin{array}{c} \frac{1}{p(t)}{\left|\Delta^{w}u_{n}\left(t\right)-\Delta^{w}u\left(t\right)\right|}^{p(t)}\leq C\left(\frac{1}{p(t)}|\Delta^{w}u_{n}{\left(t\right)|}^{p(t)}+\frac{1}{p(t)}{\left|\Delta^{w}u\left(t\right)\right|}^{p(t)}\right). \end{array}$$

Hence, functions $\frac{1}{p(t)}{\left|\Delta^{w}u_{n}\left(t\right)-\Delta^{w}u\left(t\right)\right|}^{p(t)}$ have equi-absolutely continuous integrals and from ([20], Theorem 3, p. 153), one obtains

$$\begin{array}{c} \lim_{n\to \infty }\int_{T}\frac{1}{p(t)}{\left|\Delta^{w}u_{n}\left(t\right)-\Delta^{w}u\left(t\right)\right|}^{p(t)}\Delta t=0, \end{array}$$

which implies that

$$\begin{array}{c} \lim_{n\to \infty }\int_{T}{\left|\Delta^{w}u_{n}\left(t\right)-\Delta^{w}u\left(t\right)\right|}^{p(t)}\Delta t=0. \end{array}$$

Consequently, $\Delta^{w}u_{n}\to \Delta^{w}u$ in $L^{p(t)}\left(T\right)$, which means that $u_{n}\to u$ in X. □

Now, observe that using the Minty–Browder Theorem [21] (Theorem 3.3.1, p.161) (see Lemmas 7–9), we obtain that $\varphi_{1}^{\prime }$ has an inverse mapping ${\left(\varphi_{1}^{\prime }\right)}^{-1}:X^{*}\to X$. The following holds.

**Lemma** **11.** *The operator $\varphi_{1}^{\prime }$ is a homeomorphism.*

**Proof.**  It suffices to show that ${\left(\varphi_{1}^{\prime }\right)}^{-1}$ is continuous. Let $z_{n},z\in X^{*}$, $z_{n}\to z$. Then, there are $u_{n},u\in X$, such that $\varphi_{1}^{\prime }\left(u_{n}\right)=z_{n}$ and $\varphi_{1}^{\prime }\left(u\right)=z$. Since $\varphi_{1}^{\prime }$ is continuous, $\left(u_{n}\right)$ is bounded in *X*. Without loss of generality, let $u_{n}⇀v$. Then, we have

$$\begin{array}{c} \lim_{n\to \infty }\left(\varphi_{1}^{\prime }\left(u_{n}\right)-\varphi_{1}^{\prime }\left(u\right)\right)\left(u_{n}-v\right)=\lim_{n\to \infty }\left(z_{n}\right)\left(u_{n}-v\right)=0. \end{array}$$

From Lemma 10, $u_{n}\to v$ in *X*. Consequently, $u_{n}\to u$ in *X*. □

## 5. Existence of a Solution Using the Direct Method

The direct method of the calculus of variations has a long and interesting history described in the introduction of [2] and is expressed as follows in a functional setting.

**Theorem** **1.** *Ref.* [22], p.455*. Let X be a reflexive Banach space and φ:X→R be a weakly lower semi-continuous and weakly coercive functional. Then, there exists $x_{0}\in X$, such that $\varphi \left(x_{0}\right)=\min_{x\in X}\varphi \left(x\right)$.*

Now, we give sufficient conditions for the existence of critical point of functional φ defined in (22). Consequently, by (21) and Remark 2, we prove the existence of weak solutions to the Dirichlet problem (20).

**Theorem** **2.** *If there exist $\beta \in (1,p^{-}]$, $c_{1},c_{3}\geq 0$ and*

(36) $$\begin{array}{c} c_{2}<\frac{\beta }{p^{+}\mu_{\Delta }\left(T\right)A^{\beta }} \end{array}$$

*with A given in (14), such that function F satisfies*

(37) $$F\left(t,x\right)\leq c_{1}\left|x\right|+\frac{c_{2}}{\beta }{\left|x\right|}^{\beta }+c_{3}$$

*for Δ−a.a. t∈T and x∈R, then problem (20) has a weak solution.*

**Proof.**  Our goal is to apply Theorem 1 for functional φ defined in (22). First, we shall show the coerciveness of φ. By (14), (11), (36), (37) and Theorem 6, if ${∥u∥}_{X}\to \infty$, one has

$$\begin{array}{ccc} \varphi (u) & = & \int_{T}\frac{1}{p(t)}{\left|\Delta^{w}u\left(t\right)\right|}^{p(t)}\Delta t-\int_{T}F\left(t,u^{\sigma }\left(t\right)\right)\Delta \;t\\ & \geq & \frac{1}{p^{+}}\rho_{p(\cdot )}\left(\Delta^{w}u\right)-c_{1}\int_{T}|u^{\sigma }\left(t\right)|\Delta t-\frac{c_{2}}{\beta }\int_{T}{\left|u^{\sigma }\left(t\right)\right|}^{\beta }\Delta t-c_{3}\int_{T}1\Delta \;t\\ & \geq & \frac{1}{p^{+}}∥\Delta^{w}{u∥}_{L^{p(t)}\left(T\right)}^{p^{-}}-c_{1}{∥u∥}_{T}\mu_{\Delta }\left(T\right)-\frac{c_{2}}{\beta }{∥u∥}_{T}^{\beta }\mu_{\Delta }\left(T\right)-c_{3}\mu_{\Delta }\left(T\right)\\ & \geq & \frac{1}{p^{+}}{∥u∥}_{X}^{p^{-}}-\frac{c_{2}A^{\beta }}{\beta }{∥u∥}_{X}^{\beta }\mu_{\Delta }\left(T\right)-c_{1}A{∥u∥}_{X}\mu_{\Delta }\left(T\right)-c_{3}\mu_{\Delta }\left(T\right)\to \infty , \end{array}$$

where $\rho_{p(\cdot )}$ is the modular defined in (8). Hence φ is weakly coercive over *X*.

By Lemma 5, we see that functional $\varphi_{1}$ defined in (24) is continuous. Since $s\mapsto \frac{1}{p(t)}s^{p(t)}$ is convex on [0,∞) for Δ−a.a. t∈T, $\varphi_{1}$ is convex. Consequently, $\varphi_{1}$ is weakly lower semi–continuous.

Observe that the following holds

(38) $$\begin{array}{c} \mathrm{if}\;\;u_{n}⇀u\;\;\mathrm{in}\;\;X,\;\;\mathrm{then}\;\;u_{n}^{\sigma }\to u^{\sigma }\;\;\mathrm{in}\;\;L^{p(t)}\left(T\right). \end{array}$$

Indeed, if $u_{n}⇀u$ in *X*, then ${\left(u_{n}\right)}_{n\in N}$ is bounded in *X* and, from (14), bounded in C(T). Denote by ${\left(v_{n}\right)}_{n\in N}$ a subsequence of ${\left(u_{n}\right)}_{n\in N}$. Then, since the embedding in (13) is compact, ${\left(v_{n}\right)}_{n\in N}$ has a strongly convergent subsequence. By the uniqueness of weak limit, ${\left(v_{n}\right)}_{n\in N}$ converges to *u*. Consequently, since every subsequennce ${\left(v_{n}\right)}_{n\in N}$ of ${\left(u_{n}\right)}_{n\in N}$ has a subsequence which tends to *u*, ${\left(u_{n}\right)}_{n\in N}$ converges to *u* strongly in C(T) and, from (14), $u_{n}^{\sigma }\to u^{\sigma }$ in $L^{p(t)}\left(T\right)$.

Now, using (38), Lemma 2 and proceeding similarly as in the proof of Lemma 6, one can show that $F\left(t,u_{n}^{\sigma }\left(t\right)\right)\to F\left(t,u^{\sigma }\left(t\right)\right)$ for Δ−a.a. t∈T and conclude that functional $\varphi_{2}$ defined in (25) is strongly continuous over *X*.

Consequently, φ is weakly lower semi-continuous over *X*. From Theorem 1, φ has a minimum point and the problem (20) has a weak solution. □

**Corollary** **2.** *If there exist $\beta \in (1,p^{-})$ and $c_{1},c_{2},c_{3}\geq 0$ such that function F satisfies condition (37), then problem (20) has a weak solution.*

**Proof.**  The proof is analogous to the proof of Theorem 2 with one exception. If $\beta \neq p^{-}$, then to show that φ is weakly coercive, no inequality of the type (36) is needed. □

**Remark** **3.** *Assume that the following condition holds*

(F)’ *f:T×R→R is a* Δ*–Carathéodory function over T×R.*

*Then, (F)’ together with (37) and Proposition 9 guarantee that Lemmas 5 and 6 hold.*

**Example** **1.** Notice that condition (37) is satisfied if

$$\begin{array}{c} F\left(t,x\right)=\frac{c_{2}}{\beta }{\left|x\right|}^{\beta }+G\left(t,x\right) \end{array}$$

where

$$\begin{array}{c} G\left(t,x\right)=\int_{0}^{x}g\left(t,s\right)ds \end{array}$$

and $g\left(t,x\right)\leq c_{1}$ for Δ−a.a. t∈T, x∈R. In particular, problem (20) with *f* given by

$$\begin{array}{c} f\left(t,x\right)=c_{2}{\left|x\right|}^{\beta -1}+g\left(t\right) \end{array}$$

has a weak solution for every bounded function $g\in L^{1}\left(T\right)$.

## 6. Existence of a Nontrivial Solution Using the Mountain Pass Theorem

The existence conditions of Theorem 2 are satisfied when F(t,x) does not grow too fast when x→∞. We now use another tool of the variational calculus, namely a minimax instead of a minimum characterization of a critical point of the functional, to prove the existence of a nontrivial solution of problem (20) when f(t,x) tends fast enough to 0 when x→0 (insuring the existence of the trivial solution) and fast enough to infinity when x→∞.

We say that $C^{1}$–functional φ:X→R satisfies the Palais–Smale condition, denoted (PS), if any sequence ${\left(u_{n}\right)}_{n\in N}$ in *X*, such that ${\left(\varphi \left(u_{n}\right)\right)}_{n\in N}$ is bounded and $\varphi^{\prime }\left(u_{n}\right)\to 0$ as n→∞, admits a convergent subsequence.

**Lemma** **12.** *If there exist M>0 and $\tau >p^{+}$, such that*

(39) 0<τF(t,x)≤xf(t,x)

*for Δ−a.a.t∈T and |x|≥M, then functional φ defined in (22) satisfies the (PS) condition.*

**Proof.**  Assume that ${\left(u_{n}\right)}_{n\in N}$ is a sequence such that $u_{n}\in X$ for n∈N, ${\left(\varphi \left(u_{n}\right)\right)}_{n\in N}$ is bounded and $∥\varphi^{\prime }\left(u_{n}\right){∥}_{X^{*}}\to 0$ as n→∞.

First, we shall show that ${\left(u_{n}\right)}_{n\in N}$ is bounded. Let ε>0. Since $∥\varphi^{\prime }\left(u_{n}\right){∥}_{X^{*}}\to 0$, we obtain that there exists $n_{0}\in N$, such that $∥\varphi^{\prime }\left(u_{n}\right){∥}_{X^{*}}<\varepsilon$ for $n\geq n_{0}$. Thus, we have

(40) $$\begin{array}{c} \varphi^{\prime }\left(u_{n}\right)\left(u_{n}\right)\geq -\varepsilon {∥u_{n}∥}_{X} \end{array}$$

for $n\geq n_{0}$. Moreover,

$$\begin{array}{ccc} \varphi^{\prime }\left(u_{n}\right)\left(u_{n}\right) & = & \int_{T}{\left|\Delta^{w}u_{n}\left(t\right)\right|}^{p(t)}\Delta t-\int_{T}f\left(t,u_{n}^{\sigma }\left(t\right)\right)u_{n}^{\sigma }\left(t\right)\Delta \;t\\ & = & \rho_{p(\cdot )}\left(\Delta^{w}u_{n}\right)-\int_{T}f\left(t,u_{n}^{\sigma }\left(t\right)\right)u_{n}^{\sigma }\left(t\right)\Delta t \end{array}$$

for n∈N, where $\rho_{p(\cdot )}$ is the modular defined in (8). Since *f* is the $L^{1}$–Carathéodory function over T×R, integrals

$$\begin{array}{c} \int_{T_{M_{n}}}F\left(t,u_{n}^{\sigma }\left(t\right)\right)\Delta t\;\;\;\mathrm{and}\;\;\;\int_{T_{M_{n}}}f\left(t,u_{n}^{\sigma }\left(t\right)\right)u_{n}^{\sigma }\left(t\right)\Delta t, \end{array}$$

where $T_{M_{n}}=\left\{t\in T:\right|u_{n}^{\sigma }\left(t\right)\left|<M\right\}$ are bounded. Moreover, by (39), we have

(41) $$\int_{T\backslash T_{M_{n}}}\left(f\left(t,u_{n}^{\sigma }\left(t\right)\right)u_{n}^{\sigma }\left(t\right)-\tau F\left(t,u_{n}^{\sigma }\left(t\right)\right)\right)\Delta t>0.$$

Since ${\left(\varphi \left(u_{n}\right)\right)}_{n\in N}$ is bounded, by (40) and (41), we obtain

(42) $$\begin{array}{ccc} C_{1}+\varepsilon {∥u_{n}∥}_{X} & \geq & \tau \varphi \left(u_{n}\right)-\left(\varphi^{\prime }u_{n}\right)\left(u_{n}\right)\\ \; & = & \tau \int \frac{1}{p(t)}{\left|\Delta^{w}u_{n}\left(t\right)\right|}^{p(t)}\Delta t-\tau \int_{T}F\left(t,u_{n}^{\sigma }\left(t\right)\right)\Delta t\\ \; & \; & -\rho_{p(\cdot )}\left(\Delta^{w}u_{n}\right)+\int_{T}f\left(t,u_{n}^{\sigma }\left(t\right)\right)u_{n}^{\sigma }\left(t\right)\Delta t\\ \; & \geq & \frac{\tau }{p^{+}}\int {\left|\Delta^{w}u_{n}\left(t\right)\right|}^{p(t)}\Delta t-\tau \int_{T}F\left(t,u_{n}^{\sigma }\left(t\right)\right)\Delta t\\ \; & \; & -\rho_{p(\cdot )}\left(\Delta^{w}u_{n}\right)+\int_{T}f\left(t,u_{n}^{\sigma }\left(t\right)\right)u_{n}^{\sigma }\left(t\right)\Delta t\\ \; & = & \frac{\tau }{p^{+}}\rho_{p(\cdot )}\left(\Delta^{w}u_{n}\right)-\tau \int_{T}F\left(t,u_{n}^{\sigma }\left(t\right)\right)\Delta t\\ \; & \; & -\rho_{p(\cdot )}\left(\Delta^{w}u_{n}\right)+\int_{T}f\left(t,u_{n}^{\sigma }\left(t\right)\right)u_{n}^{\sigma }\left(t\right)\Delta t\\ \; & = & \left(\frac{\tau }{p^{+}}-1\right)\rho_{p(\cdot )}\left(\Delta^{w}u_{n}\right)\\ \; & \; & +\int_{T}\left(f\left(t,u_{n}^{\sigma }\left(t\right)\right)u_{n}^{\sigma }\left(t\right)-\tau F\left(t,u_{n}^{\sigma }\left(t\right)\right)\right)\Delta t \end{array}$$

with $C_{1}\in R$ and $n\geq n_{0}$. By (11), (41) and Proposition 6, we have

(43) $$\begin{array}{c} \left(\frac{\tau }{p^{+}}-1\right)\rho_{p(\cdot )}\left(\Delta^{w}u_{n}\right)+\int_{T}\left(f\left(t,u_{n}^{\sigma }\left(t\right)\right)u_{n}^{\sigma }\left(t\right)-\tau F\left(t,u_{n}^{\sigma }\left(t\right)\right)\right)\Delta \;t\\ \geq \left(\frac{\tau }{p^{+}}-1\right)\min\left\{∥u_{n}{∥}_{X}^{p^{+}},{∥u_{n}∥}_{X}^{p^{-}}\right\}+C_{2} \end{array}$$

with $C_{2}\in R$ and $n\geq n_{0}$. By (42) and (43), the following assertion holds

$$\begin{array}{ccc} C_{1}+\varepsilon {∥u_{n}∥}_{X} & \geq & \left(\frac{\tau }{p^{+}}-1\right)\min\left\{∥u_{n}{∥}_{X}^{p^{+}},{∥u_{n}∥}_{X}^{p^{-}}\right\}+C_{2} \end{array}$$

with $C_{1},C_{2}\in R$. Hence, we obtain

$$\begin{array}{c} \left(\frac{\tau }{p^{+}}-1\right)\min\left\{∥u_{n}{∥}_{X}^{p^{+}},{∥u_{n}∥}_{X}^{p^{-}}\right\}-\varepsilon {∥u_{n}∥}_{X}\leq C_{3} \end{array}$$

with $C_{3}\in R$. Since $\tau >p^{+}$, ${\left(∥u_{n}{∥}_{X}\right)}_{n\in N}$ is bounded.

Now, without loss of generality, we assume that $u_{n}⇀u$ in *X*. Using the same arguments as in the proof of Theorem 2, one can show that for $\varphi_{2}^{\prime }:X\to X^{*}$ we have: $u_{n}⇀u$ implies $\varphi_{2}^{\prime }\left(u_{n}\right)\to \varphi_{2}^{\prime }\left(u\right)$. Since

$$\begin{array}{c} \varphi^{\prime }\left(u_{n}\right)=\varphi_{1}^{\prime }\left(u_{n}\right)-\varphi_{2}^{\prime }\left(u_{n}\right)\to 0\;\mathrm{as}\;n\to \infty , \end{array}$$

we obtain that $\varphi_{1}^{\prime }\left(u_{n}\right)\to \varphi_{2}^{\prime }\left(u\right)$. Hence, using Lemma 11, $u_{n}\to u$ in *X*. Consequently, φ satisfies (PS) condition. □

The existence of nontrivial solutions to problem (20) will be shown using the Mountain Pass Theorem of Ambrosetti and Rabinowitz [23], which we recall here in the following form.

**Theorem** **3.** *Ref.* [24]* (p.7). Let X be a Banach space and let φ:X→R be a $C^{1}$–functional satisfying (PS) condition. Suppose that φ(0)=0 and*

*(i)*  *there are constants $\alpha_{0},r_{0}>0$, such that $\varphi {|}_{{∥e∥}_{X}=r_{0}}\geq \alpha_{0}$;*
*(ii)*  *there is an element $e_{0}\in X$, such that $∥e_{0}{∥}_{X}>r_{0}$ and $\varphi (e_{0})\leq 0$.*

*Then functional φ has a critical point with critical value $c_{0}\geq \alpha_{0}$ characterized by*

$$c_{0}=\underset{\gamma \in \Gamma }{\inf}\max_{u\in \gamma ([0,1])}\varphi \left(u\right),$$

*where $\Gamma =\{\gamma \in C\left(\left[0,1\right],X\right):\gamma \left(0\right)=\theta_{X}\;\mathrm{and}\;\gamma \left(1\right)=e_{0}\}$.*

**Theorem** **4.** *If condition (39) is satisfied and*

(44) $$\lim_{u\to 0}\frac{f(t,u)}{{\left|u\right|}^{p^{+}-1}}=0$$

*uniformly with respect to u for Δ−a.a. t∈T, then problem (20) has a nontrivial weak solution.*

**Proof.**  First, observe that φ is unbounded from below, i.e., for any ξ>0, there is an element e∈X with ${∥e∥}_{X}\geq \xi$, such that φ(e)<0. Let us denote

$$\begin{array}{c} T_{\alpha M}=\{t\in T:|\alpha u^{\sigma }\left(t\right)\left|\geq M\right\} \end{array}$$

for α≥1 and u∈X. We will show that if u∈X is such that $\mu_{\Delta }\left(T_{1M}\right)>0$, then φ(αu)→−∞ as α→∞. For α≥1, we see that $T_{1M}\subset T_{\alpha M}$ and hence, $\mu_{\Delta }\left(T_{\alpha M}\right)>0$.

By (39), we have

(45) $$\frac{f(t,u)}{F(t,u)}\geq \frac{\tau }{u}$$

for u≥M and

(46) $$\frac{f(t,u)}{F(t,u)}\leq \frac{\tau }{u}$$

for u≤−M and with $\tau >p^{+}$. Integrating both sides of (45), we obtain

$$F\left(t,u\right)\geq \frac{F(t,M)}{M^{\tau }}u^{\tau }$$

for Δ–a.a. t∈T and u≥M. Similarly, by (46), we have

$$F\left(t,u\right)\geq \frac{F(t,-M)}{M^{\tau }}{\left(-u\right)}^{\tau }$$

for Δ–a.a. t∈T and u≤−M. Consequently, there exists function $\omega \in L^{1}\left(T\right)$, such that

(47) $$F\left(t,u\right)\geq {\omega \left(t\right)|u|}^{\tau }$$

for Δ–a.a. t∈T and |u|≥M.

Let $u_{0}\in X$. For α>1, we have

(48) $$\begin{array}{ccc} \varphi (\alpha u_{0}) & = & \int_{T}\frac{1}{p(t)}{\left|\alpha \Delta^{w}u_{0}\right|}^{p(t)}\Delta t-\int_{T}F\left(t,\alpha u_{0}^{\sigma }\left(t\right)\right)\Delta t\\ & \leq & \frac{\alpha^{p^{+}}}{p^{-}}\rho_{p(\cdot )}\left(\Delta^{w}u_{0}\right)-\int_{\left\{t\in T:|\alpha u_{0}^{\sigma }\left(t\right)|<M\right\}}F\left(t,\alpha u_{0}^{\sigma }\left(t\right)\right)\Delta t\\ & & -\int_{\left\{t\in T:|\alpha u_{0}^{\sigma }\left(t\right)|\geq M\right\}}F\left(t,\alpha u_{0}^{\sigma }\left(t\right)\right)\Delta t, \end{array}$$

where $\rho_{p(\cdot )}$ is the modular defined in (8). By (47), we obtain

$$\begin{array}{c} F\left(t,\alpha u_{0}^{\sigma }\left(t\right)\right)\geq \omega \left(t\right)\alpha^{\tau }{\left|u_{0}\left(t\right)\right|}^{\tau } \end{array}$$

for t∈T, such that $|\alpha u_{0}^{\sigma }\left(t\right)|\geq M$. Thus,

(49) $$\begin{array}{ccc} \int_{\left\{t\in T:|\alpha u_{0}^{\sigma }\left(t\right)|\geq M\right\}}F\left(t,\alpha u_{0}^{\sigma }\left(t\right)\right)\Delta t & \geq & \alpha^{\tau }\int_{\left\{t\in T:|\alpha u_{0}^{\sigma }\left(t\right)|\geq M\right\}}\omega \left(t\right){\left|u_{0}\left(t\right)\right|}^{\tau }\Delta t\\ \; & = & \alpha^{\tau }K_{1}\left(u_{0}\right) \end{array}$$

with $K_{1}\left(u_{0}\right)>0$. Moreover, by assumption (F1), we have

(50) $$\begin{array}{c} \int_{\left\{t\in T:|u_{0}^{\sigma }\left(t\right)|<M\right\}}\left|F\left(t,\alpha u_{0}^{\sigma }\left(t\right)\right)\right|\Delta t\leq K_{2} \end{array}$$

with $K_{2}>0$. By (48), (49) and (50), we estimate

$$\begin{array}{ccc} \varphi (\alpha u_{0}) & \leq & \frac{\alpha^{p^{+}}}{p^{-}}\rho_{p(\cdot )}\left(\Delta^{w}u_{0}\right)-\int_{\left\{t\in T:|\alpha u_{0}^{\sigma }\left(t\right)|<M\right\}}F\left(t,\alpha u_{0}^{\sigma }\left(t\right)\right)\Delta \;t\\ & & -\int_{\left\{t\in T:|\alpha u_{0}^{\sigma }\left(t\right)|\geq M\right\}}F\left(t,\alpha u_{0}^{\sigma }\left(t\right)\right)\Delta \;t\\ & \leq & \frac{\alpha^{p^{+}}}{p^{-}}\rho_{p(\cdot )}\left(\Delta^{w}u_{0}\right)-\alpha^{\tau }K_{1}\left(u_{0}\right)+K_{2}. \end{array}$$

It implies that $\varphi (\alpha u_{0})\to -\infty$ as α→∞, since $\tau >p^{+}$ and $u_{0}$ is fixed.

Now, we shall show that there exist constants $\alpha_{0},r_{0}>0$ such that $\varphi {|}_{{∥u∥}_{X}=r_{0}}\geq \alpha_{0}$. By (23) and (44), for every ε>0 there exists δ>0, such that

(51) $$\begin{array}{c} F\left(t,u\right)\leq \frac{\varepsilon }{p^{+}}{\left|u\right|}^{p^{+}} \end{array}$$

for Δ−a.a. t∈T and |u|<δ. Let $\varepsilon_{0}\in \left(0,\frac{1}{A^{p^{+}}\mu_{\Delta }\left(T\right)}\right)$. Then, by (6) and (51), there is $\delta_{1}>0$, such that

(52) $$\begin{array}{ccc} \int_{\left\{t\in T:|u^{\sigma }\left(t\right)|<\delta_{1}\right\}}F\left(t,u^{\sigma }\left(t\right)\right)\Delta t & \leq & \frac{\varepsilon_{0}}{p^{+}}\int_{T}|u^{\sigma }{\left(t\right)|}^{p^{+}}\Delta t\leq \frac{\varepsilon_{0}}{p^{+}}{∥u^{\sigma }∥}_{T}^{p^{+}}\mu_{\Delta }\left(T\right)\\ & \leq & \frac{\varepsilon_{0}}{p^{+}}A^{p^{+}}{∥u∥}_{X}^{p^{+}}\mu_{\Delta }\left(T\right) \end{array}$$

with *A* defined in (14).

Let $\delta_{0}<\min\left\{\delta_{1},A\right\}$ and ${∥u∥}_{X}=r_{0}\in \left(0,\frac{\delta_{0}}{A}\right]$. Then,

(53) $$\begin{array}{c} {∥u∥}_{X}\leq \frac{\delta_{0}}{A}\leq 1. \end{array}$$

By (14),

(54) $$\begin{array}{c} ∥u^{\sigma }{∥}_{T}\leq {∥u∥}_{T}\leq A{∥u∥}_{X}\leq A\frac{\delta_{0}}{A}=\delta_{0}. \end{array}$$

Combining (52) with (54), we conclude that

$$\begin{array}{c} \int_{T}F\left(t,u^{\sigma }\left(t\right)\right)\Delta t=\int_{\left\{t\in T:|u^{\sigma }\left(t\right)|<\delta_{1}\right\}}F\left(t,u^{\sigma }\left(t\right)\right)\Delta t\leq \frac{\varepsilon_{0}}{p^{+}}A^{p^{+}}{∥u∥}_{X}^{p^{+}}\mu_{\Delta }\left(T\right) \end{array}$$

and therefore, from (53) and Proposition 6, one has

$$\begin{array}{ccc} \varphi (u) & = & \int_{T}\frac{1}{p(t)}{\left|\Delta^{w}u\left(t\right)\right|}^{p(t)}\Delta t-\int_{T}F\left(t,u^{\sigma }\left(t\right)\right)\Delta t\\ & \geq & \frac{1}{p^{+}}\int_{T}|\Delta^{w}{u\left(t\right)|}^{p(t)}\Delta t-\frac{\varepsilon_{0}}{p^{+}}A^{p^{+}}\mu_{\Delta }\left(T\right){∥u∥}_{X}^{p^{+}}\\ & = & \frac{1}{p^{+}}\rho_{p(\cdot )}\left(\Delta^{w}u\right)-\frac{\varepsilon_{0}}{p^{+}}A^{p^{+}}\mu_{\Delta }\left(T\right){∥u∥}_{X}^{p^{+}}\\ & \geq & \frac{1}{p^{+}}{∥u∥}_{X}^{p^{+}}-\frac{\varepsilon_{0}}{p^{+}}A^{p^{+}}\mu_{\Delta }\left(T\right){∥u∥}_{X}^{p^{+}}\\ & = & \frac{1}{p^{+}}\left(1-\varepsilon_{0}A^{p^{+}}\mu_{\Delta }\left(T\right)\right){∥u∥}_{X}^{p^{+}}. \end{array}$$

Consequently, we obtain that there exists $\alpha_{0}>0$, such that $\varphi {|}_{{∥u∥}_{X}=r_{0}}\geq \alpha_{0}>0$. Since $\varphi (\theta_{X})=0$, the statement follows from Theorem 3. □

**Example** **2.** Consider problem (20) with the function *f* defined as

$$\begin{array}{c} f\left(t,x\right)=h\left(t\right)x^{\alpha } \end{array}$$

for t∈T, x∈R, where $h\in L^{1}\left(T\right)$, α is an odd number and $\alpha >p^{+}$. One can easily check that Assumption (44) holds. Moreover, for τ:=α+1 and M>0, Assumption (39) is satisfied.

## 7. Conclusions

Using direct variational methods and the mountain pass theorem, we have obtained several sufficient conditions for the existence of solutions to the p(t)–Laplacian Dirichlet problem on a bounded time scale. Some results regarding the regularity of solutions have also been included in this paper. We have shown that a sort of unification in discrete and continuous settings is possible with the use of a time-scale notion.

## Data Availability

Not applicable.
